# Race and Mortality

**Published:** 1902-12-13

**Authors:** 


### RACE AND MORTALITY.

The question, How far race affects mortality? is one of much interest, for if any serious difference can be shown to exist in the stamina or power of resistance to disease possessed by the different races of mankind, the nationality of the patient becomes a matter of the first importance in regard both to treatment and prognosis. In certain of the United States, where the population is divided into two very definite sections by a rigid line of colour, this problem is a tempting one, and seems almost to ask for investigation. In a recent number of the *Medical Examiner*, an American paper largely devoted to matters of life-insurance, the whole problem is very carefully considered by F. J. Hoffman, statistician to the Prudential Insurance Company, who comes to the conclusion that "the various races and types of mankind differ fundamentally and widely in longevity and specific disease liability." Turning to the census reports on vital statistics, he shows that at every age-period the mortality among the coloured is far higher than among the whites. Thus "it would be the height of folly to grant equal rates to races of such radically different degrees of vital resistance." Many other tables are given teaching the same lesson. Thus it is shown that the death-rate from consumption among coloured adults is nearly four times as great as among whites, while it is nine times as great among coloured as among white children. Summing up the matter we are told that "evidently the facts prove that race and nativity materially modify the rate of mortality and the special disease liability."

On looking carefully, however, into the many interesting tables given, we find room for doubting whether the data do in fact prove the existence of special liability to disease in the negro as such. That the death-rate among the coloured population is excessive there seems to be no doubt or question. But before we can put this down to any special weakness of stamina, any special defect in disease-resisting power, we must be assured that the average negro is exposed to no greater risks, and that the sanitary conditions amid which he lives are on an average no worse than those of the surrounding whites; and it is here that the statistics not only fail but appear to suggest another explanation. Among the tables given is one which as well as showing the comparative mortalities of whites and blacks, gives also the comparative mortalities of various races of whites, arranged according to the nationalities of their mothers. From this we see that while the difference in the death-rates between whites and coloured far transcends any that is to be found between the whites of various nativities, yet a very considerable difference does occur among the several groups of whites. At adult ages it is the Irish who, next to the coloured races, have the highest death-rate. Take the age-period of 34 to 44, and we find that, against 7·5 among the United States whites, it was 15·0—that is, more than twice as great—among the Irish, and 21·0, or nearly three times as great, among the coloured population; while in regard to consumption the Irish death-rate at certain ages treads closely on the heels of the excessive mortality of the coloured races. What is there, then, which is common to the coloured and the Irish?

In regard to the difference between the death-rates of the native-born and the foreign whites, we are told that "The fact must not be overlooked that the foreign whites are very largely employed in dangerous and unhealthy occupations . . . so that the observed difference in the death-rate is probably less the result of type than of social and economic condition." Doos not this quotation let the cat out of the bag? Is it not the fact that the very influences which lead to excessive mortality among the foreign-born, and especially the Irish, in American cities, operate in an enhanced degree among the coloured races. The Irish who emigrate are to a large extent unskilled labourers who live in poverty amid insanitary conditions. Hence they die. But is not this tenfold the case with the coloured races in the South, and do they not also die for the same reason? This is the point on which the whole question hinges. At first sight the reading of the statistics seems to teach us that when we see a sick negro we must allow for an inherent deficiency of stamina and give a prognosis proportionately grave because he is a negro; and, indeed, that for other races also we must make a "correction" for nationality before we can truly estimate their vital resistance. A more careful reading seems to show that certain races are willing to accept, and are even capable of being happy among, surrouudings which to others would be absolutely repugnant; and that the striking differences in mortality are to be explained by differences in mode of life and in sanitary surroundings rather than by some inexplicable racial tendency to disease.

